# Automated measurement of upper thermal limits in small aquatic animals

**DOI:** 10.1242/jeb.182386

**Published:** 2018-09-13

**Authors:** Tim Burton, Bettina Zeis, Sigurd Einum

**Affiliations:** 1 Centre for Biodiversity Dynamics, Department of Biology, Norwegian University of Science and Technology, Realfagbygget, NO-7491 Trondheim, Norway; 2 Institut für Zoophysiologie, Westfälische Wilhelms-Universität, Hindenburgplatz 55, D-48143 Münster, Germany

**Keywords:** Thermal tolerance, Computer vision, Critical thermal limits, Temperature stress

## Abstract

We present a method for automating the measurement of upper thermal limits in small aquatic organisms. Upper thermal limits are frequently defined by the cessation of movement at high temperature, with measurement being performed by manual observation. Consequently, estimates of upper thermal limits may be subject to error and bias, both within and among observers. Our method utilises video-based tracking software to record the movement of individuals when exposed to high, lethal temperatures. We develop an algorithm in the R computing language that can objectively identify the loss of locomotory function from tracking data. Using independent experimental data, we validate our approach by demonstrating the expected response in upper thermal limits to acclimation temperature.

## Abstract (precis)

**Summary:** We present a method for the automated measurement of upper thermal tolerance in small aquatic organisms from video data.

## INTRODUCTION

Physiological limits are emerging as a key predictor of organismal sensitivity to environmental change (Huey et al., 2012). Physiological limits can be exemplified by the concept of the thermal performance curve, which typically shows how a physiological variable, but also a life history trait or population vital rate, varies in relation to temperature (Sinclair et al., 2016). These performance curves, and their spatial and temporal variation, are useful for understanding how variation in a given trait can influence the sensitivity of a genotype, population or species to temperature (e.g. Chown et al., 2010). Consequently, in the face of global environmental change, renewed attention has been given to the thermal sensitivity of ‘performance’, and in particular, measurements of the upper boundary on the thermal performance curve (referred to hereafter as the upper thermal limit or UTL), as well as the application of these physiological measures to ecologically relevant questions. For example, UTLs have been used to investigate phenotypic and evolutionary responses to temperature change (Brans et al., 2017; Geerts et al., 2015; Manenti et al., 2014; Phillips et al., 2016; Sandblom et al., 2016; Yampolsky et al., 2014), to understand how species distributions are shaped by the environment (Clusella-Trullas et al., 2011; Kellermann et al., 2012; Overgaard et al., 2014), and to understand how niches and distributions might respond to environmental change (Kearney et al., 2009; Nowakowski et al., 2018). From a methodological perspective, UTLs can be measured in a multitude of ways (summarised by Angilletta, 2009; Terblanche et al., 2011). However, with the exception of direct estimates of survival following thermal stress (e.g. LT_50_), measurements of UTLs typically involve recording the time to loss of locomotory function following exposure to lethal temperature (e.g. knock-down time) or the temperature at which locomotory function is lost (e.g. knock-down temperature or CT_max_). These measurements are typically performed by manual observation, with the observer noting either the time or the temperature at which the experimental animals lose locomotory function. It has previously been recognised that such measures of UTLs are subjective and thus prone to error and bias (Lighton and Turner, 2004), both within and among observers. As such, alternative methods that overcome these issues have been developed (e.g. thermolimit respirometry; Lighton and Turner, 2004), but they are data intensive, requiring simultaneous measurement of individual oxygen consumption and activity data. However, in developing their method, Lighton and Turner (2004) concluded that UTLs can be likely be assessed from individual movement data alone. Here, we describe an automated, high-throughput system for measuring UTLs from movement data. Our system utilises video-based tracking software that simultaneously records the movement of multiple individuals when exposed to high, lethal temperature. We implement an algorithm in R that uses the tracking data to find optimal and objective criteria for detecting the loss of locomotory function at high temperature. To illustrate the utility of our method, we apply these criteria to an independent experimental dataset.

## MATERIALS AND METHODS

We developed our method using the clonally reproducing zooplankter *Daphnia magna* Straus 1820 as a study organism. We measured the UTL in this species as the ability to maintain mobility at high temperature, recorded as the ‘time to immobilisation’ (*T*_imm_) at a temperature 37°C, which is lethal after several hours of exposure (Yampolsky et al., 2014). Motility is an established parameter for measuring physiological performance in *D. magna* (Knie, 1982; Yampolsky et al., 2014; Zeis et al., 2004), and given its small body size (approximate body length at maturity is ∼2 mm), our measurement temperature likely reflected the internal body temperature of the organism. Our method can be summarised in four steps. First, we experimentally induced among-individual variation in *T*_imm_ through acclimation to different temperatures (e.g. Cambronero et al., 2017; Moyano et al., 2017). Second, when exposed to 37°C, we filmed the experimental individuals, up to and including the point when they lost swimming function (i.e. became immobile). Third, using animal tracking software, we obtained a time-series of movement data for these individuals. Fourth, we developed a custom R script (https://www.r-project.org/) in conjunction with a quantitative statistical approach, to objectively identify a threshold movement value that could define a state of immobility from the tracking data. Each of these steps is described in further detail below.

### Step 1: use acclimation to induce variation in UTLs

Developmentally synchronised clonal animals were obtained by isolating neonates (*F*_0_) produced within a 24 h period from mass cultures of the experimental clone (EF 64, hatched from an ephippium collected at Værøy Island, northern Norway, 67.687°N, 12.672°E) that we maintain in our laboratory at 17°C. *F*_0_ individuals and two subsequent generations (*F*_1_ and *F*_2_), both founded from second-brood offspring, were reared at either 17°C or 22°C in 2.5 l aquaria in temperature-controlled climate cabinets (IPP260, Memmert, Schwabach, Germany), a long-day photoperiod (16 h:8 h light:dark) and initial density of 35 females per aquarium. Culture medium [artificial *Daphnia* medium (ADaM)] was replaced in the aquaria one to two times per week and commercial shellfish diet (1800, Reed Mariculture, Campbell, CA, USA) was provided at *ad libitum* levels three times per week. We predicted that individuals from the 22°C acclimation treatment would have a higher *T*_imm_ than individuals from the 17°C treatment (Cambronero et al., 2017; Yampolsky et al., 2014).

### Step 2: film experimental animals whilst exposed to high temperature

**Fig. 1.**
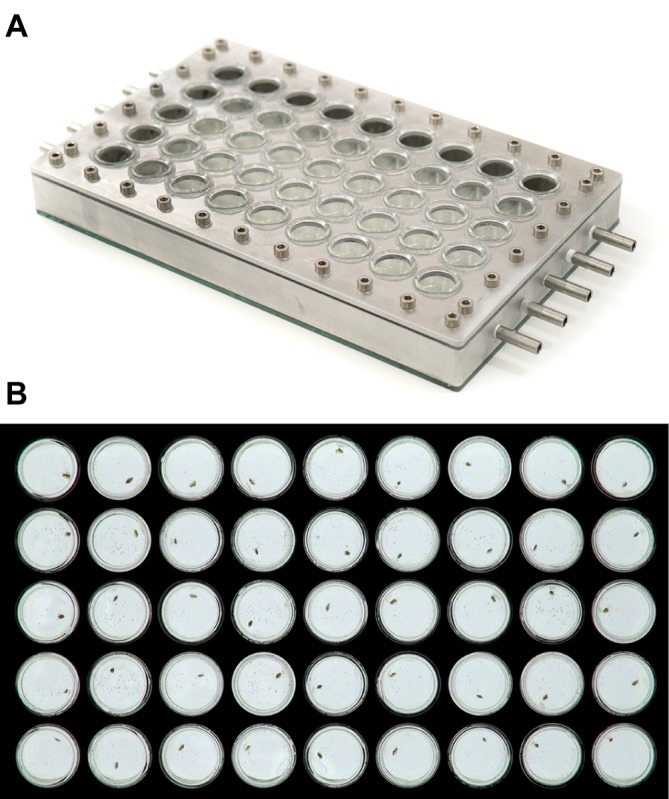
**Thermostatic well plate.** (A) Image of thermostatic well-plate used to measure time to immobilisation (*T*_imm_) in *Daphnia magna*. Water, heated to the desired measuring temperature, is circulated through the well plate via the five inlet and outlet points located on the short sides of the plate. Each well is open on its upper surface, facilitating transfer of experimental individuals in and out of the well plate. Image credit: Fredrik Jutfelt. (B) Image of well plate showing an individual daphnid in each well. Optimal contrast for tracking is obtained by placing the well plate on top of an LED light board. Each well is 16 mm in diameter.

We exposed individual daphnids from each of the acclimation treatments to lethally high temperature using a custom-built aluminium and glass thermostatic well plate (Fig. 1). Forty-five individual glass wells, open on their upper surface (well diameter 16 mm, depth 21 mm), were inserted in a rectangular 5×9 array on an aluminium plate (length 265 mm, width 125 mm, thickness 3 mm). This plate was fitted (and sealed via a series of screws) on top of a rectangular aluminium frame (depth 25 mm, length 265 mm, width 125 mm, thickness 20 mm). A sheet of glass (thickness 3 mm) of the same dimensions as the aluminium frame was glued to its underside. Water, warmed to 37°C (in a 15.0 l capacity water bath, Grant Instruments, Shepreth, UK), was pumped into the water-jacket via five inlet points (Eheim Compact 600 pump, Deizisau, Germany). The wells were positioned in the plate so that there was a space of approximately 3 mm between the bottom of each well and the floor of the well plate, and a space of approximately 4 mm between adjacent wells and the outer walls of the well plate. This means that water could pass freely from the inlet points, both around and beneath each well, to five outlet points located at the opposite end of the plate. Water then flowed back to the water bath. Pilot measurements confirmed that there was no difference in temperature of medium contained in wells at the periphery or centre of the well plate, nor between wells located immediately adjacent to the inlet or outlet points. The temperature of medium in each well also reflected the set temperature of the water bath.

*F*_2_ animals from each of the acclimation treatments were measured approximately 1, 2, 3, 6, 8, 10 and 13 days after reaching maturity (determined as presence of first clutch eggs in brood pouch). On a given recording day, we filmed *n*=45 individuals for a unique combination of acclimation treatment and age group. Thus, the 17°C and 22°C acclimation treatments were measured in separate ‘blocks’ and the respective age groups within each block were recorded as sequence of ‘runs’ beginning with the 1 day group on the first day and concluding with the final age group 13 days later. On a given recording day, up to 45 min before filming began, *F*_2_ females from two randomly chosen aquaria of the same acclimation treatment were pooled, mixed and placed in fresh ADaM (without food) of the same temperature as the treatment group from which they originated. Forty-five of these females were pipetted individually into plastic vials along with 3.3 ml of ADaM. These vials were then returned to their climate cabinet until filming began. The 45 individuals from an acclimation group (and medium of the same temperature) were transferred into the wells (i.e. one individual per well), which had been pre-heated to the test temperature by circulating medium from the water bath, noting down the well number and time (in seconds) elapsed from the moment that the first individual was placed in a well (on average, it took 3–4 min to introduce all 45 individuals to the well plate). Video recording commenced after the last individual was introduced to a well. The well plate was backlit with an LED light board (Huion A4 LED light pad, set to maximum intensity; Shenzen, China), to provide contrast between the individual in each well and the background. The well plates were filmed from above with digital video cameras (Basler aCA1300-60gm, fitted with 5–50 mm, F1.4, CS mount lenses; Ahrensburg, Germany) at a working distance of 116 cm (from lens to upper surface of well plate), a frame rate of 30 frames s^−1^ and a resolution of 1280×960 pixels. Video recording ceased when visual inspection indicated that all individuals were motionless (inspection performed ca. every 2 min). As the animals began to lose swimming ability during video recording, we did not physically stimulate them because of possible interference with subsequent tracking. Thus, the thermal limit identified by our method may differ slightly from one defined by the loss of reaction to mild physical stimulus at the chosen measurement temperature (e.g. gentle prodding). After filming, all animals were photographed under a stereomicroscope, and body size (carapace length, mm) was measured with ImageJ software (version 1.49v, National Institutes of Health, Bethesda, MD, USA). In total, we filmed 315 individuals from each of the acclimation treatments (*n*=630 total).

### Step 3: process video file with animal tracking software

The video files produced in step 2 were processed in Ethovision (version XT 11.5, Noldus Information Technology, Wageningen, The Netherlands; settings: greyscale pixel range 0–120, pixel size range 2–130, sample rate 3 observations s^−1^), to produce a time series of velocity data (in mm s^−1^, travelled by the center-point of each individual).

### Step 4: quantitatively define a state of immobility

We developed an R script based upon a moving median (med.filter function from robfilter package; https://cran.r-project.org/web/packages/robfilter/) that calculates the time taken for an individual's swimming velocity to drop below a specified threshold value (*V*_thresh_). Moving medians are frequently used to detect underlying ‘signals’ or patterns from ‘noisy’ time-series data, such as the tracking data utilised here. The moving median requires the parameter *w*, which specifies the window width, the number of observations in a time series over which each successive iteration of the moving median is calculated. Given that an individual's *T*_imm_ should be positively affected by warm acclimation temperatures (Cambronero et al., 2017; Moyano et al., 2017) and should be lower in larger, older individuals (Brans et al., 2017; Messmer et al., 2017), the optimal choice of values for *V*_thresh_ and *w* should be the combination that maximises the amount of variation in *T*_imm_ that can be described by acclimation treatment (17 and 22°C), when also statistically controlling for the variation in body size that was present both among and within each acclimation treatment (see Fig. S1).

To find these optimal values, we used the velocity time-series data for 629 of the individuals produced in step 3 (one animal was misplaced during removal from the well plate) to calculate *T*_imm_ for 5400 unique combinations of *V*_thresh_ (0.01 to 1 in increments of 0.01 mm s^−1^) and *w* (20 to 1080 in increments of 20 observations, corresponding to window widths that ranged from 6.67 to 360 s given that the movement of each individual was tracked at a rate of 3 observations s^−1^). The upper limits of both *V*_thresh_ and *w* were chosen based upon preliminary tests that revealed that values larger or smaller than these produced estimates of *T*_imm_ of which little variation could be attributed to acclimation treatment. For each combination of *V*_thresh_ and *w*, the estimated *T*_imm_ of each individual was modelled statistically as a function of acclimation treatment and its body size (general linear model, temperature treatment and body size as additive categorical and continuous predictors, respectively). This procedure was also repeated for the interactive version of the same model.

To test the optimal values for *V*_thresh_ and *w*, we applied them to an independent set of time-series swimming velocity data. The same *D. magna* genotype used to define optimal values for *V*_thresh_ and *w* was reared at a wider range of acclimation temperatures (12, 17, 20, 24 and 28°C) and nine individuals from each treatment group were tested for *T*_imm_ using the same procedure described in step 2, but in a single well-plate ‘run’. Developmentally synchronised clonal animals were obtained by isolating neonates produced within a 24 h period from mass cultures of the same genotype described earlier in the Materials and Methods (EF 64). These individuals were reared in 250 ml jars at the five different temperatures (nine replicate jars per temperature) in temperature-controlled climate cabinets (Memmert IP260) at a long-day photoperiod (16 h:8 h light:dark) and a density of five individuals per jar. A commercial shellfish diet (1800, Reed Mariculture) was provided at *ad libitum* levels three times per week. Culture medium (ADaM; Klüttgen et al., 1994) in the jars was changed one to two times per week. The establishment of these cultures was staggered, with the lower temperatures established earlier so that all individuals were measured for *T*_imm_ at a similar developmental stage (i.e. when all were carrying first clutch offspring in the brood pouch). Up to 45 min before video recording in the well plate commenced, nine mature females from each acclimation treatment (one per jar) were placed individually into plastic vials along with 3.5 ml of fresh ADaM (without food) of the same temperature as the treatment group from which they originated (i.e. 12°C to 28°C). These vials were then returned to their respective climate cabinets until recording began. Again we anticipated a positive relationship between *T*_imm_ and acclimation temperature. Moreover, our hypothesis was that the *V*_thresh_ and *w* values that maximised the amount of variation in *T*_imm_ that could be explained by acclimation temperature (and body size) in the design employed in Step 2 should be as, if not more, effective in a situation in which estimates of *T*_imm_ should be free of variation attributable to ‘run’ and ‘block’ effects.

## RESULTS AND DISCUSSION

**Fig. 2.**
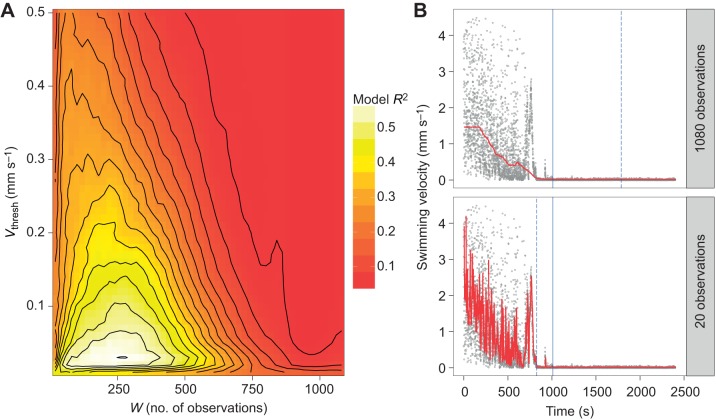
**Determining optimal criteria for objective estimation of upper thermal limits from video data.** (A) Contour plot showing amount of variation, *R*^2^, in *T*_imm_ explained by acclimation temperature (17°C versus 22°C) in relation to window width (*w*) and threshold swimming velocity (*V*_thresh_). Values of *V*_thresh_ and *w* that maximised the amount of variation in *T*_imm_ that could be described by acclimation temperature were 0.03 mm s^−1^ and 280 observations, respectively. To facilitate interpretation, *V*_thresh_ values greater than 0.5 were excluded from the figure. See Materials and Methods for full details of the analysis. (B) Scatter plots showing time series of swimming velocity data for individual 621. The effect of the widest (*w*=1080 observations) versus narrowest window widths (*w*=20 observations) tested on estimation of the moving median are depicted by the red line in the upper and lower panels respectively. In each panel, the solid blue line indicates *T*_imm_ as estimated for individual 621 by the optimal combination of *V*_thresh_ and *w* revealed in A, whereas the dashed blue line indicates *T*_imm_ as estimated for the optimal *V*_thresh_ value, but for a *w* of either 1080 or 20 observations (upper and lower panels, respectively).

**Fig. 3.**
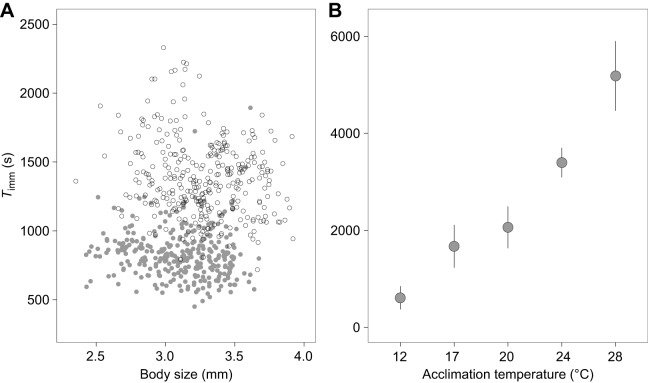
***T*_imm_ estimated by automated method.** (A) *T*_imm_ plotted against body size for the individuals used to determine optimal values for *V*_thresh_ and *w*. Animals were acclimated to either 17°C (grey circles, *n*=315) or 22°C (white circles, *n*=314). *T*_imm_ for each individual was calculated using the optimal values for *V*_thresh_ and *w* identified in Fig. 2A. (B) Mean *T*_imm_ estimates (±95% confidence interval) produced by the optimal combination of *V*_thresh_ and *w* for individuals that had been acclimated to 12, 17, 20, 24 or 28°C (*n*=9 per temperature).

For each of the 5400 combinations of *V*_thresh_ and *w* that were tested, the amount of variation in *T*_imm_ that could be explained by acclimation treatment and body size was maximised for *V*_thresh_=0.03 mm s^−1^ and *w*=280 observations (Fig. 2A). When this procedure was performed for the interactive version of the same general linear model (i.e. including body size×acclimation treatment interaction), it yielded the same result. Fig. 2A also reveals how the choice of *w* had greater influence on the estimation of *T*_imm_ than *V*_thresh_. Fig. 2B demonstrates the effect of different *w* values on the estimation of *T*_imm_: relatively wide *w* values were less sensitive to the variation inherent in a given time series and vice versa for relatively narrow *w* value. Consequently, for a given *V*_thresh_, a relatively wide *w* was more likely to produce estimates of *T*_imm_ that were higher than those produced by the optimal *w* and vice versa for a relatively narrow *w* (Fig. 2B). The optimal values for *V*_thresh_ and *w* produced *T*_imm_ estimates that matched our predictions with respect to acclimation temperature and body size: *T*_imm_ was higher in individuals that had been acclimated to 22°C and decreased with body size/age (parameter estimates±s.e. and corresponding *t*-values for 22°C acclimated individuals compared with 17°C acclimated individuals: 566.58±19.23, *t*=29.50, *P*<0.0001; body size: −114.97±32.27, *t*=−3.56, *P*<0.001; model *R*^2^=0.59; Fig. 3A). Moreover, when applying the optimal combination of parameter values to the independent tracking data obtained from five different acclimation temperatures (12–28°C, *n*=9 individuals per acclimation temperature), *T*_imm_ increased consistently with acclimation temperature, with the latter explaining 88.7% of the total variation in *T*_imm_ (Fig. 3B).

We view the method and hardware described here as a general framework that end-users can adapt to objectively measure UTLs in their own study organism(s), but caution that the optimal parameters (*V*_thresh_ and *w*) for identifying UTLs described here are unlikely to be generalizable across study organisms/taxa. Our R script and hardware was developed to measure UTLs in (a) a relatively small, (b) aquatic organism at a (c) static test temperature. However, provided that the focal organism can be filmed and subsequently tracked accurately whilst exposed to warming, our system could be adapted to measure larger or smaller study organisms (both aquatic and terrestrial) by scaling and adjusting the hardware accordingly. Furthermore, via the addition of ‘real-time’ temperature data, our system could also incorporate the ramped changes in test temperature that are employed in other measurements of UTLs, such as CT_max_. Nevertheless, our method is unlikely to be applicable to all species. A prerequisite for the successful translation of our method is that the study organism/life stage in question is motile and maintains a certain level of spontaneous activity. The physiological UTL may also be difficult to establish based on our method if the study organism becomes immobile as part of its natural response to an increase in ambient temperature. However, having said that, estimating and explaining variation in the ‘voluntary’ immobility response to thermal heating may be equally interesting owing to the consequences this can have on ecological processes such as feeding rates and predation risk.

In summary, we present an alternative means to eliminate observer subjectivity when estimating UTLs. In comparison with other established techniques such as thermolimit respirometry (Lighton and Turner, 2004), our method can more efficiently facilitate the measurement of larger sample sizes, meaning that weaker biological effects can be identified within shorter periods of time. We acknowledge that a possible criticism of our technique is that it employs proprietary tracking software. We chose the tracking software described here based on its long history in animal behaviour research and its relative ease of use. There are, however, several open-source alternatives that are more coding intensive but perform similar tracking functions (summarised in Robie et al., 2017). Provided these open-source programmes are able to track the focal organisms accurately, we see no reason as to why our algorithm would fail to produce transferrable results. Moreover, driven by rapid developments in the application of computer vision methods to address ecological questions (Weinstein, 2017) and, in particular, automated, quantitative analysis of behaviour, we feel that this number of computer vision methods will undoubtedly increase in the near future, making our method more accessible to a broader range of end-users.

## Supplementary Material

Supplementary information

## References

[JEB182386C1] Angilletta M. J. (2009). *Thermal Adaptation: a Theoretical and Empirical Synthesis*. Oxford: Oxford University Press.

[JEB182386C2] Brans K. I., Jansen M., Vanoverbeke J., Tüzün N., Stoks R. and De Meester L. (2017). The heat is on: genetic adaptation to urbanization mediated by thermal tolerance and body size. *Glob. Change Biol.* 23, 5218-5227. 10.1111/gcb.13784 28614592

[JEB182386C3] Cambronero M. C., Zeis B. and Orsini L. (2017). Haemoglobin-mediated response to hyper-thermal stress in the keystone species *Daphnia magna*. *Evol. Appl.* 11, 112-120. 10.1111/eva.12561 29302276 PMC5748520

[JEB182386C4] Chown S. L., Hoffmann A. A., Kristensen T. N., Angilletta M. J. Jr, Stenseth N. C. and Pertoldi C. (2010). Adapting to climate change: a perspective from evolutionary physiology. *Clim. Res.* 43, 3-15. 10.3354/cr00879

[JEB182386C5] Clusella-Trullas S., Blackburn T. M. and Chown S. L. (2011). Climatic predictors of temperature performance curve parameters in ectotherms imply complex responses to climate change. *Am. Nat.* 177, 738-751. 10.1086/660021 21597251

[JEB182386C7] Geerts A. N., Vanoverbeke J., Vanschoenwinkel B., Van Doorslaer W., Feuchtmayr H., Atkinson D., Moss B., Davidson T. A., Sayer C. D. and De Meester L. (2015). Rapid evolution of thermal tolerance in the water flea *Daphnia*. *Nat. Clim. Change* 5, 665-668. 10.1038/nclimate2628

[JEB182386C8] Huey R. B., Kearney M. R., Krockenberger A., Holtum J. A. M., Jess M. and Williams S. E. (2012). Predicting organismal vulnerability to climate warming: roles of behaviour, physiology and adaptation. *Philos. Trans. R. Soc. B Biol. Sci.* 367, 1665-1679. 10.1098/rstb.2012.0005 PMC3350654 22566674

[JEB182386C9] Kearney M. R., Shine R. and Porter W. P. (2009). The potential for behavioral thermoregulation to buffer “cold-blooded” animals against climate warming. *Proc. Natl Acad. Sci. USA* 106, 3835-3840. 10.1073/pnas.0808913106 19234117 PMC2656166

[JEB182386C10] Kellermann V., Overgaard J., Hoffmann A. A., Fløjgaard C., Svenning J.-C. and Loeschcke V. (2012). Upper thermal limits of *Drosophila* are linked to species distributions and strongly constrained phylogenetically. *Proc. Natl Acad. Sci. USA* 109, 16228-16233. 10.1073/pnas.1207553109 22988106 PMC3479592

[JEB182386C11] Klüttgen B., Dülmer U., Engels M. and Ratte H. T. (1994). ADaM, an artificial freshwater for the culture of zooplankton. *Water Res.* 28, 743-746. 10.1016/0043-1354(94)90157-0

[JEB182386C12] Knie J. (1982). Der Daphnientest. *Decheniana* 26, 82-86.

[JEB182386C13] Lighton J. R. B. and Turner R. J. (2004). Thermolimit respirometry: an objective assessment of critical thermal maxima in two sympatric desert harvester ants, *Pogonomyrex rugosus* and *P. californicus*. *J. Exp. Biol.* 207, 1903-1913. 10.1242/jeb.00970 15107444

[JEB182386C14] Manenti T., Sørensen J. G., Moghadam N. N. and Loeschke V. (2014). Predictability rather than amplitude of temperature fluctuations determines stress resistance in a natural population of *Drosophila simulans*. *J. Evol. Biol.* 27, 2113-2122. 10.1111/jeb.12463 25146297

[JEB182386C15] Messmer V., Pratchett M. S., Hoey A. S., Tobin A. J., Coker D. J., Cooke S. J. and Clark T. D. (2017). Global warming may disproportionately affect larger adults in a predatory coral reef fish. *Glob. Change Biol.* 23, 2230-2240. 10.1111/gcb.13552 27809393

[JEB182386C16] Moyano M., Candebat C., Ruhbaum Y., Álvarez-Fernández S., Claireaux G., Zambonino-Infante J.-L. and Peck M. A. (2017). Effects of warming rate, acclimation temperature and ontogeny on the critical thermal maximum of temperate marine fish larvae. *PLoS ONE* 12, e0179928 10.1371/journal.pone.0179928 28749960 PMC5531428

[JEB182386C17] Nowakowski A. J., Watling J. I., Thompson M. E., Brusch G. A., Catenazzi A., Whitfield S. M., Kurz D. J., Suárez-Mayorga Á., Aponte-Gutiérrez A., Donnelly M. A. et al. (2018). Thermal biology mediates responses of amphibians and reptiles to habitat modification. *Ecol. Lett.* 21, 345-355. 10.1111/ele.12901 29314479

[JEB182386C18] Overgaard J., Kearney M. R. and Hoffmann A. A. (2014). Sensitivity to thermal extremes in Australian *Drosophila* implies similar impacts of climate change on the distribution of widespread and tropical species. *Glob. Change Biol.* 20, 1738-1750. 10.1111/gcb.12521 24549716

[JEB182386C19] Phillips B. L., Muñoz M. M., Hatcher A., Macdonald S. L., Llewelyn J., Lucy V. and Moritz C. (2016). Heat hardening in a tropical lizard: geographic variation explained by the predictability and variance in environmental temperatures. *Funct. Ecol.* 30, 1161-1168. 10.1111/1365-2435.12609

[JEB182386C20] Robie A. A., Seagraves K. M., Egnor S. E. R. and Branson K. (2017). Machine vision methods for analyzing social interactions. *J. Exp. Biol.* 220, 25-34. 10.1242/jeb.142281 28057825

[JEB182386C21] Sandblom E., Clark T. D., Gräns A., Ekström A., Brijs J., Sundström L. F., Odelström A., Adill A., Aho T. and Jutfelt F. (2016). Physiological constraints to climate warming in fish follow principles of plastic floors and concrete ceilings. *Nat. Commun.* 7, 11447 10.1038/ncomms11447 27186890 PMC4873662

[JEB182386C22] Sinclair B. J., Marshall K. E., Sewell M. A., Levesque D. L., Willett C. S., Slotsbo S., Dong Y., Harley C. D. G., Marshall D. J., Helmuth B. S. et al. (2016). Can we predict ectotherm responses to climate change using thermal performance curves and body temperatures? *Ecol. Lett.* 19, 1372-1385. 10.1111/ele.12686 27667778

[JEB182386C23] Terblanche J. S., Hoffmann A. A., Mitchell K. A., Rako L., le Roux P. C. and Chown S. L. (2011). Ecologically relevant measures of tolerance to potentially lethal temperatures. *J. Exp. Biol.* 214, 3713-3725. 10.1242/jeb.061283 22031735

[JEB182386C24] Weinstein B. (2017). A computer vision for animal ecology. *J. Anim. Ecol.* 7, 11447. 10.1111/1365-2656.12780 29111567

[JEB182386C25] Yampolsky L. Y., Schaer T. M. M. and Ebert D. (2014). Adaptive phenotypic plasticity and local adaptation for temperature tolerance in freshwater zooplankton. *Proc. R. Soc. B Biol. Sci.* 281, 20132744 10.1098/rspb.2013.2744 PMC3871322 24352948

[JEB182386C26] Zeis B., Maurer J., Pinkhaus O., Bongartz E. and Paul R. J. (2004). A swimming activity assay shows that the thermal tolerance of *Daphnia magna* is influenced by temperature acclimation. *Can. J. Zool.* 82, 1605-1613. 10.1139/z04-141

