# Supplementary material for: Automated measurement of upper thermal limits in small aquatic animals
Source: J Exp Biol. 2018 Sep 13;221(17):jeb182386. doi: 10.1242/jeb.182386 (PMC6140313; doi:10.1242/jeb.182386)
Supplement: Supplementary information [file jexbio-221-182386-s1.pdf]

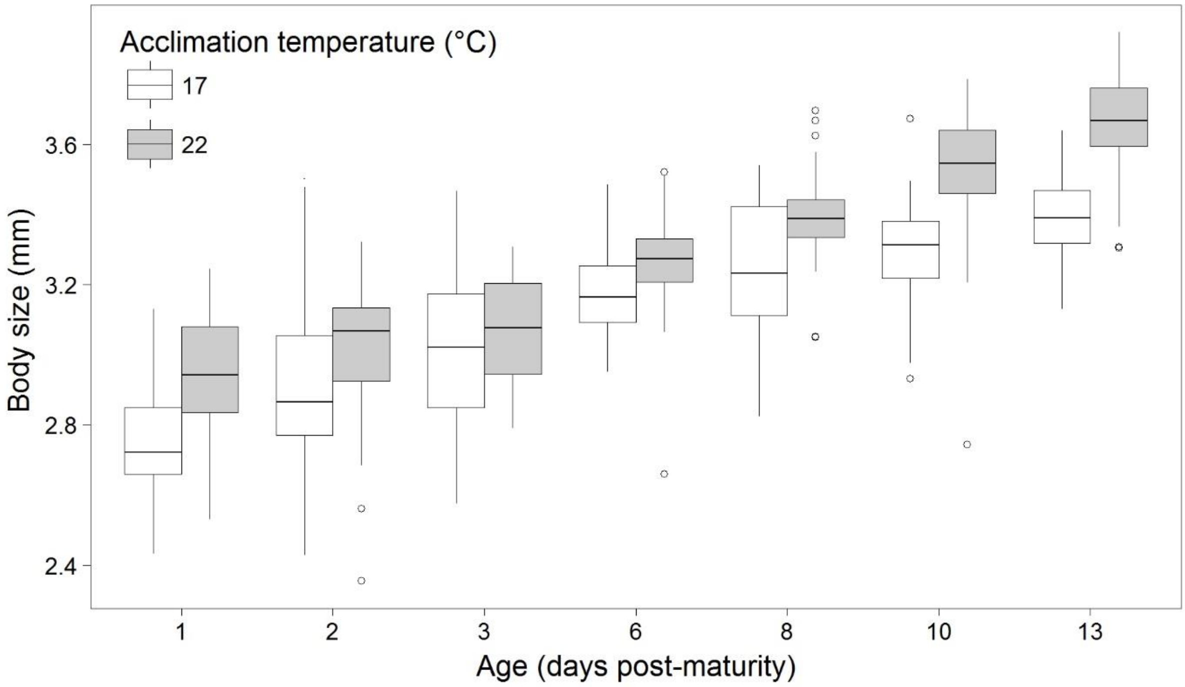

Fig. S1. Boxplot showing variation in body size plotted against age (expressed as number of days elapsed since eggs were first evident in the brood pouch) for *Daphnia magna* that had been acclimated to either 17 (white boxes) or 22°C (grey boxes). The interquartile range is represented by the top and bottom of each box and the median by the centreline. The whiskers extend up to 1.5 times the interquartile range from the extremes of the box to the furthest datum within that distance. Any data beyond that distance are presented as possible 'outliers', indicated by circles.

Journal of Experimental Biology • Supplementary information
